# Effects of Ocean Acidification on the Ballast of Surface Aggregates Sinking through the Twilight Zone

**DOI:** 10.1371/journal.pone.0050865

**Published:** 2012-12-18

**Authors:** Pedro A. de Jesus Mendes, Laurenz Thomsen

**Affiliations:** OceanLab, Jacobs University Bremen gGmbH, Bremen, Germany; University of California, Merced, United States of America

## Abstract

The dissolution of CaCO_3_ is one of the ways ocean acidification can, potentially, greatly affect the ballast of aggregates. A diminution of the ballast could reduce the settling speed of aggregates, resulting in a change in the carbon flux to the deep sea. This would mean lower amounts of more refractory organic matter reaching the ocean floor. This work aimed to determine the effect of ocean acidification on the ballast of sinking surface aggregates. Our hypothesis was that the decrease of pH will increase the dissolution of particulate inorganic carbon ballasting the aggregates, consequently reducing their settling velocity and increasing their residence time in the upper twilight zone. Using a new methodology for simulation of aggregate settling, our results suggest that future pCO_2_ conditions can significantly change the ballast composition of sinking aggregates. The change in aggregate composition had an effect on the size distribution of the aggregates, with a shift to smaller aggregates. A change also occurred in the settling velocity of the particles, which would lead to a higher residence time in the water column, where they could be continuously degraded. In the environment, such an effect would result in a reduction of the carbon flux to the deep-sea. This reduction would impact those benthic communities, which rely on the vertical flow of carbon as primary source of energy.

## Introduction

Since the beginning of the industrial revolution, the ocean has absorbed roughly half of the anthropogenic carbon dioxide released by the burning of fossil fuels. The dissolution of CO_2_ in the ocean leads to an increase in the concentrations of carbon dioxide, carbonic acid, hydrogen ions and bicarbonate ions, and to a decrease in carbonate ions. An increase in hydrogen ions will, by definition, result in a decrease in pH [1]. Since atmospheric CO_2_ concentrations continue to increase, their effect on the carbon cycle, and consequently on marine ecosystems, becomes a pressing question. However, the pH of the ocean is naturally very variable, and some authors argue that the projected changes in ocean pH will have a negligible impact on non-calcifying marine microorganisms [2].

Nonetheless, even relatively small changes in physiology of such microorganisms can result in shifts in marine biochemical cycling of elements [3]. Adding the probable and significant changes in the physiology of calcifying marine organisms, ocean acidification may cause appreciable shifts in biochemical cycling mediated by microorganisms.

Any shifts in the biochemical cycling of organic carbon, and its subsequent transfer to the deeper ocean, are of great importance. It is directly related to atmospheric CO_2_ concentrations, and deep-sea ecosystems are largely dependent on it as a source of energy.

Atmospheric CO_2_ content is increasing at an alarming rate, with projections pointing to partial pressures of 1100 µatm (or roughly three times the value in the year 2000) in the first decade of the next century [4]. However, long term projections in climate sciences have been in general conservative [5], so it is possible that these values will occur sooner. Also, as stated above, the pH of the ocean is naturally variable, so shifts to such high partial pressures might occur sooner than the correspondent increase in the atmosphere, and on vast areas of the ocean.

Sinking aggregates are a key component of the biological pump of the oceans, transporting organic matter from the photic zone to deeper waters [6]. A considerable part of the aquatic primary production is removed from the surface through coagulation processes and sedimentation of aggregates [7], [8], [9]. Aggregates are hotspots of heterotrophic activity, and they are continually consumed either by their own microbial community or by the free-living microbial communities they encounter throughout their descent [10], [11].

The settling velocity of these aggregates will determine the amount and quality of organic matter that survives remineralization during the descent [12], [13]. This will be mainly determined by the ballast of the aggregates, be it biogenic or lithogenic.

It has been recognized that the POC:ballast ratio has quite constant values throughout the water column [12]. This ratio varies for the more important ballasts: silica, carbonates and dust, and the flux of these can account for up to 90% of the POC flux to the deep sea when they are all taken into account in the same multiple regression analysis. In comparison, individual linear regressions account at most for 60% of this flux [13]. They have also shown that the transport efficiencies of the different ballasts did not vary significantly after 1000 m. This suggests that the processes shaping the aggregates occur above this depth, the so called twilight zone.

Ocean acidification has the potential to severely affect the ballast of aggregates, mainly due to the dissolution of CaCO_3_. This would reduce the settling speed of aggregates and result in a change in the carbon flux to the deep sea, with lower amounts of less bioavailable organic matter reaching the ocean floor. CaCO_3_ can have a lithogenic origin, due to weathering of rocks or resuspension of CaCO_3_-rich sediment [14], and/or a biogenic origin, mainly via calcifying marine microbes and the recently discovered fish excreted carbonates [15].

This work aimed to determine the effect of ocean acidification on the ballast of surface aggregates. Our hypothesis was that the decrease of pH will increase the dissolution of particulate inorganic carbon ballasting the aggregates, consequently reducing their settling velocity and increasing their residence time in the upper twilight zone. We have verified that such a decrease in settling velocity occurs under a high concentration of CO_2_. The implications of this decrease are discussed.

## Materials and Methods

### Model Aggregates

Since the flux of POC is so closely related to the three more important ballasts identified by Klaas and Archer (carbonate, kaolinite and smectite) [13], all three were incorporated in our experiments, to more precisely mimic the naturally occurring particles. Hamm [16] studied the effects of different lithogenic material in the aggregation and sedimentation of different diatoms. He used up to 100 mg^.^l^−1^ of individual materials, and confirmed that lithogenic particles aggregate efficiently with POC and may significantly increase the sinking rate of the produced aggregates. Although his work did not include a mix of the more important ballasts, it supplied a range of concentrations within which our aggregation experiments could be carried out. Model aggregates were produced in a Couette chamber similar in design to that of Drapeau et al. [17]. A culture of *Thalassiosira weissflogii* (OD 0.8) was incubated under a shear rate of 0.7 s^−1^ with a mix of 25 mg^.^l^−1^ of carbonate, 25 mg^.^l^−1^ of kaolinite and 25 mg^.^l^−1^ of smectite. This produced aggregates that were small and resilient enough to be used in our experimental setup, while retaining the three more important ballasts identified by Klaas and Archer [13].

### Settling Microcosm

**Figure 1:**
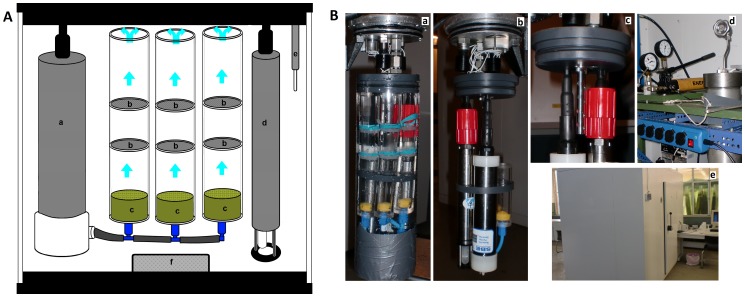
The settling microcosm used in our experiment; A: Diagram showing the components of the settling microcosm: a) pump, b) 63 µm mesh delimiting the experimental volume, c) collimators to regulate the water flow, d) p<h sensor, e) CO_2_ sensor, f) flexible membrane to equalize the pressure; 1 B: a) closed settling microcosm, connected to the lid of the pressure chamber, with the experimental tubes in first plan, b) detail of the settling microcosms, with the pH sensor and recirculation pump in first plan, c) detail of the settling microcosms, with the CO_2_ sensor in first plan, d) closed pressure chamber, e) refrigerated chamber.

A new chamber was designed for the simulation of sinking aggregates at different settling velocities, the settling microcosm. Inside the chamber were three experimental cylinders, a pump and two sensors (Fig. 1). The three cylinders had in their central section an experimental volume, delimited below and above by a 63 µm mesh. The pump inside the experimental chamber creates an upward fluid flow throughout the cylinders, to simulate the direction of flow experienced by the particles as they sink through a natural environment. This is a variation on the concept of Plough and Jorgensen [18] in which model aggregates were suspended in an upward flow mediated by a nylon mesh. The carbonate chemistry was controlled in real time by a CO_2_ sensor (Microelectrodes Inc.) and a pH sensor (AMT GmbH). The CO_2_ sensor had a range up to 9000 µatm. The pH sensor had a measuring range between 0 and 14 pH. The settling microcosms and the sensors therein can be used at pressures up to 60 MPa. The settling microcosm was placed inside a pressure chamber, its pump and sensors connected to the outside control and readout systems through SubConn underwater connectors. The whole setup was kept in a refrigerated chamber to keep it at the appropriate temperature (±0.3°C).

### Carbonate Chemistry Perturbation

The carbonate chemistry of the water used in the experiment was altered by chemical manipulation [19]. Conditions expected in future oceans of 1100 µatm were achieved by addition of 0.1 M HCl, 0.001 M Na_2_CO_3_ and 0.1 M NaHCO_3_ to the filtered artificial seawater. The volumes of the chemicals added were calculated using the seacarb package for R [20], [21]. The carbonate chemistry was altered in the closed experimental chamber to prevent gas exchange. Measurements of pH and CO_2_ concentration confirmed that the carbonate system was altered as planned. Samples were prepared and processed following the “Guides to Best Practices for Ocean CO_2_ Measurements” [22].

### Incubation Setup

The aggregates were placed inside the settling microcosm. The microcosm was filled with GF/F filtered, artificial seawater (32 psu) with a carbonate chemistry adjusted to the present or future conditions. This reduced the biological activity to the community originally present in the aggregates. The microcosm was continuously pressurized at a rate of 30 MPa/day, until it reached 10 MPa, the equivalent to a depth of 1000 m. The full incubation lasted 80 h. The experiment was triplicated at a pCO_2_ of 380 µatm and 1100 µatm. Additional incubations of similar duration were done at atmospheric pressure, with triplicates at a pCO_2_ of 380 µatm and 1100 µatm. These aimed at isolating the effect of the increase in hydrostatic pressure.

### Determination of Organic and Inorganic Carbon Content

Aggregate samples were concentrated onto precombusted Whatman GF/F glass filters and analyzed for organic carbon [23]. The filters were dried at 60°C overnight. After weighting, a section of each filter was placed into a silver cup and decarbonated with 1 M HCl to determine the organic carbon content. The samples were dried at 60°C and the process repeated until bubbling stopped. The cups were closed and compacted into spheres. These samples were analyzed with a Euro-EA Elemental analyzer (Hekatech) standardized with acetanilide. The inorganic carbon content was determined from the difference between the total carbon content and the organic carbon content.

### Determination of Particle Size and Settling Velocity (ws)

A Laser In Situ Scattering and Transmissiometry device (LISST-100X) was used to measure the variations in particle size distribution of the model aggregates before and after the incubations. The particle size vs. settling velocity relationship of phytodetrital aggregates was investigated by using a settling column of square cross-section [24]. The particles were back-illuminated and recorded with a digital video camera (Imageworks DFK-41F02) for determination of settling rates and particle sizes. The camera was capable of resolving particles of >11 µm diameter. The analysis of the particle sizes and settling velocities was done using the ImageJ (v.1.61) software. The resulting settling speeds were converted into m day^−1^ velocities, and the average speed for each of the aggregate size classes was calculated for the equivalent size classes of the LISST analysis (>63 µm, >75 µm, >88 µm, >104 µm, >122 µm, >144 µm, >170 µm, >201 µm, >237 µm, >280 µm, >331 µm, >390 µm, >460 µm).

### Statistical Analysis

The raw data were plotted (median with standard deviations) for presentation. For statistical analysis the data were standardized using the quotient of the values after the incubation (Post-Inc) by the values before the incubation (Pre-Inc).

The normality of the data was assessed with the Shapiro-Wilk test. A two-way ANOVA was performed to determine the statistical significance of the effects of pressure, pCO_2_, and the combination of both on the POC and PIC data.

For the w_s_ and size data a multivariate general linear model was used to determine the statistical significance of the effects of pressure, pCO_2_, and the combination of both on the different aggregate size classes.

All the statistical analyses were performed using the software SPSS20 (IBM).

## Results

### PIC and POC

**Figure 2:**
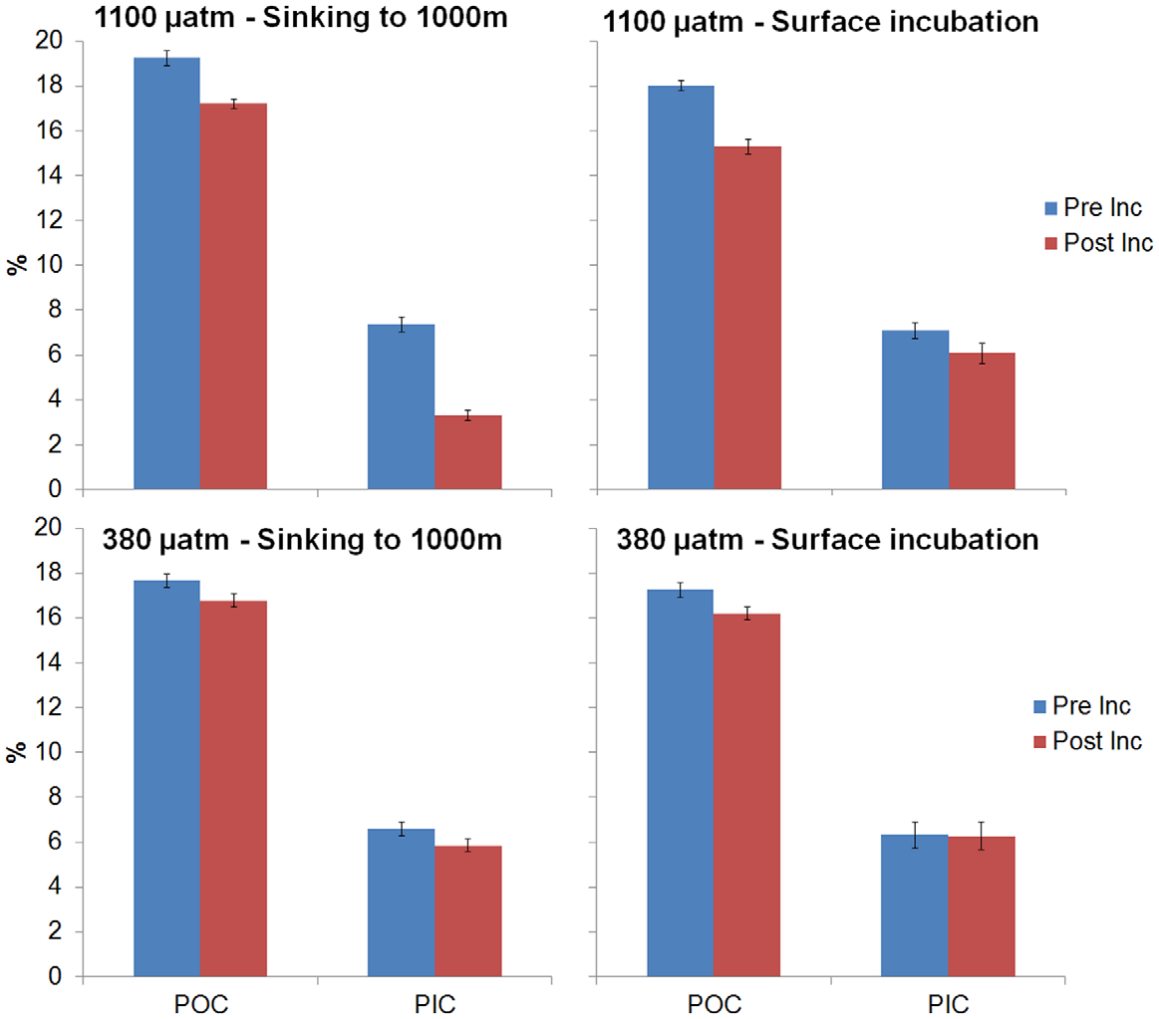
Variation of the percentages of POC and PIC before (Pre-Inc) and after the incubation (Post-Inc), for all the treatments. The horizontal bars show the standard deviation.

A significant decrease of particulate inorganic carbon (PIC) occurred under both the pCO_2_ conditions tested during the pressurized treatments, being most pronounced at 1100 µatm (Fig. 2). PIC score was normally distributed for all group combinations of pCO_2_ and pressure, as assessed by the Shapiro-Wilk test (p>0.05). There was homogeneity of variances, as assessed by the Levene Test of Homogeneity of Variance (p = 0.383). There was a statistically significant effect of both pCO_2_ (p<0.001) and pressure (p<0.001) on the PIC decrease. Under atmospheric pressure the variation of PIC was less pronounced. There was a statistically significant interaction between pCO_2_ and pressure, F(1,8) = 297, p<0.001, partial η2 = 0.974. Under future ocean conditions of 1100 µatm the pressurized treatment led to the loss of almost 50% of the CaCO_3_ ballast in the aggregates.

There was a significant decrease of particulate organic carbon (POC), in all treatments (Fig. 2). POC score was normally distributed for all group combinations of pCO_2_ and pressure, as assessed by the Shapiro-Wilk test (p>0.05). There was homogeneity of variances, as assessed by the Levene Test of Homogeneity of Variance (p = 0.164). There was a statistically significant effect of both pCO2 (p<0.001) and pressure (p<0.001). There was a higher loss of POC under future ocean conditions when compared to present conditions. There was a statistically significant interaction between pCO_2_ and pressure (p = 0.009) that seems to decrease the POC degradation. The pressurized treatments showed a lower loss of POC than their unpressurized counterparts (Fig. 2).

### Particle Size

**Figure 3:**
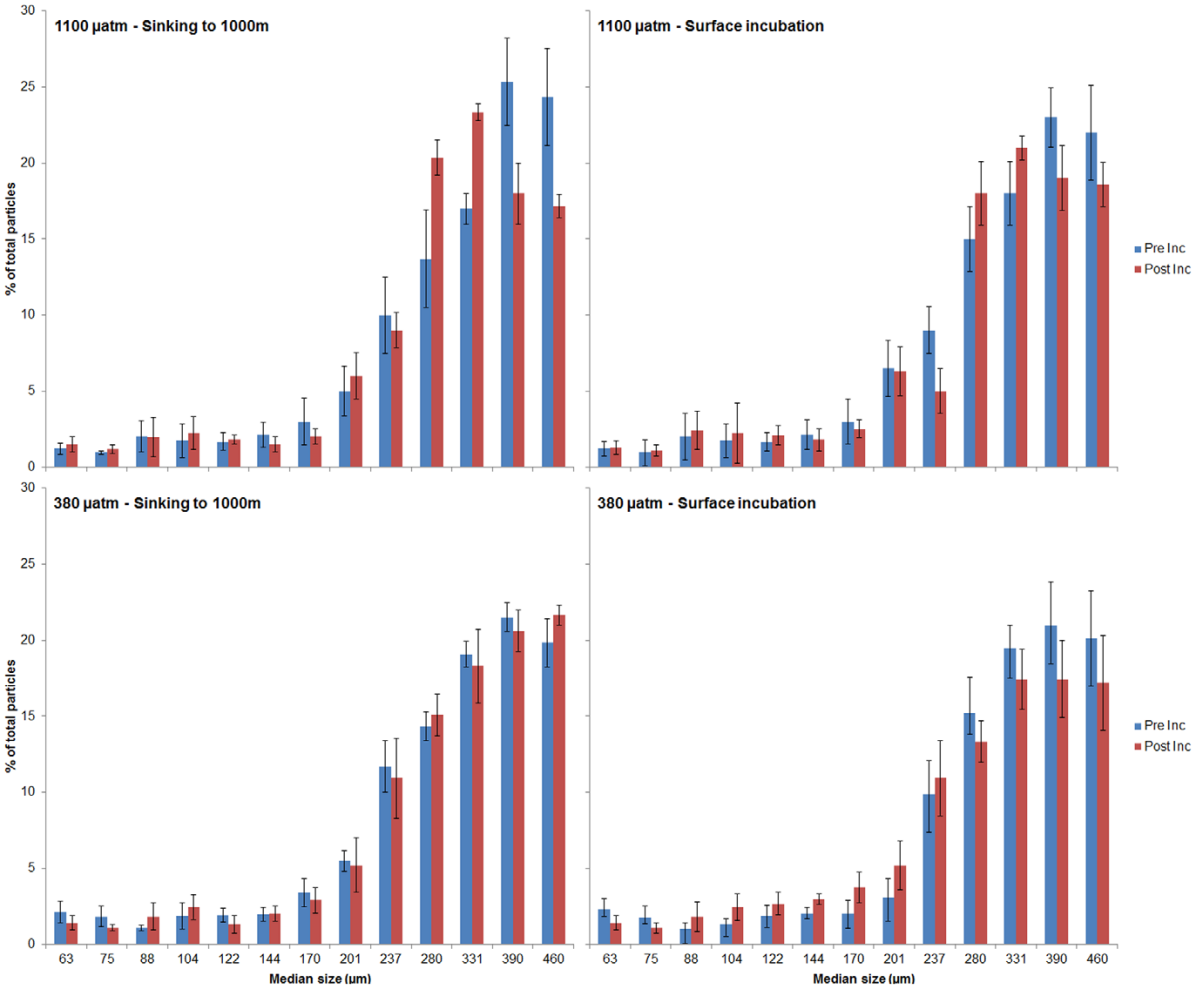
Variation of particle size before (Pre-Inc) and after the incubation (Post-Inc), for all the treatments. The horizontal bars show the standard deviation.

There was significant variation in particle size (Fig. 3) for the separate size classes. The particle size score was normally distributed for all group combinations of pCO_2_ and pressure, as assessed by the Shapiro-Wilk test (p>0.05). There was homogeneity of variances, as assessed by the Levene Test of Homogeneity of Variance (p>0.05). Under simulated future pCO_2_ conditions of 1100 µatm, but only during the pressurized treatment, there was a significant shift from larger aggregates to smaller ones. There was a decrease of aggregate abundance in the >390 and >460 µm size classes, while the >280 and >331 µm sized aggregates increased in numbers. These four size classes comprised more than 75% of the aggregate numbers. Pressure had a significant effect on this number variation in 3 of the 4 largest size classes (>460 µm, p = 0.023; >331 µm, p = 0.004; >280 µm, p = 0.031). The interaction of pressure and pCO2 only had a significant effect on the largest size class (>460 µm, p = 0.037).

### Settling Velocity (w_s_)

**Figure 4:**
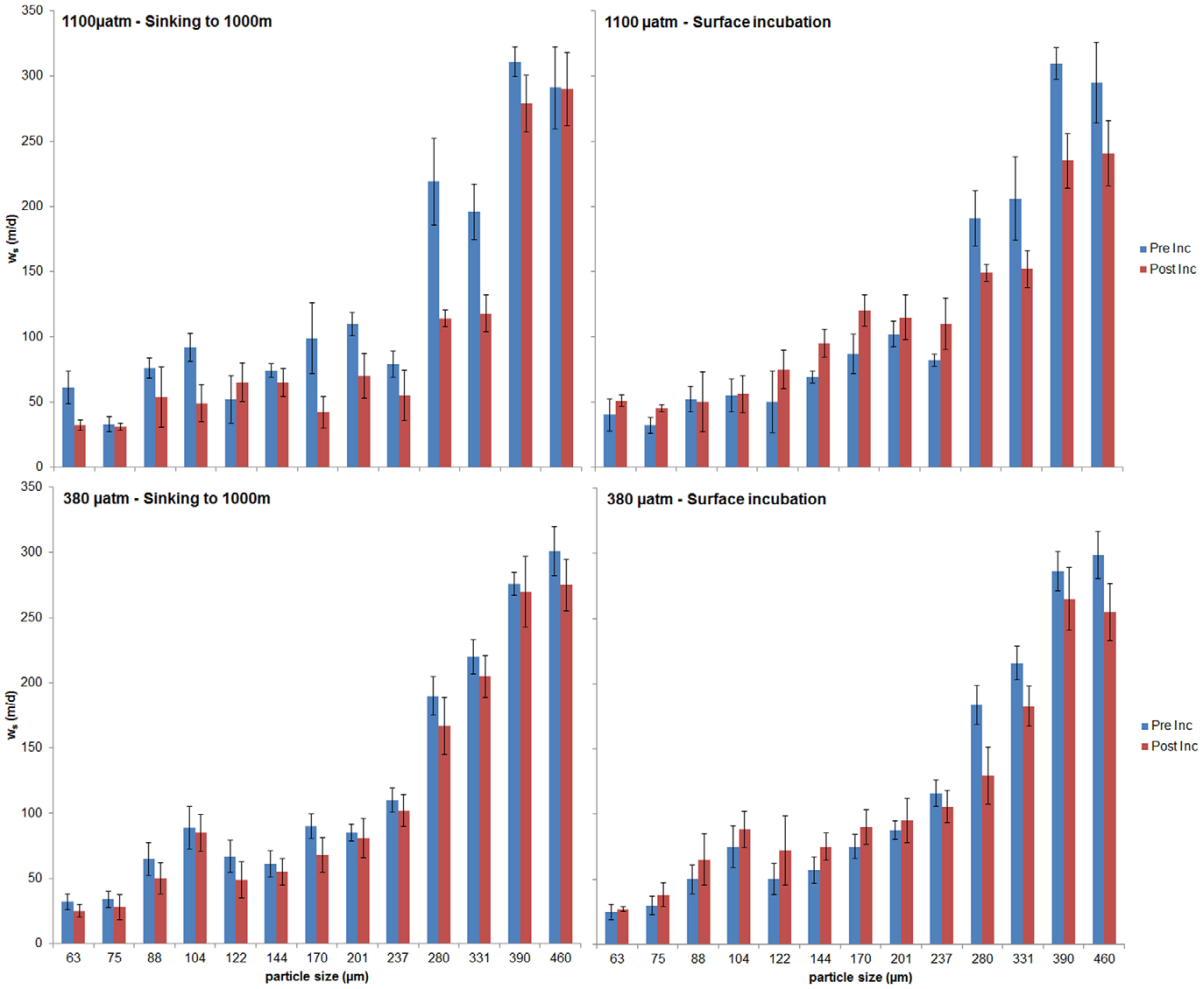
Variation of settling velocity per particle size before (Pre-Inc) and after the incubation (Post-Inc), for all treatments. The horizontal bars show the standard deviation.

In the sinking simulations, under both present and future pCO_2_ conditions, the aggregate settling velocities (w_s_) decreased in virtually all the size classes (Fig. 4). In the atmospheric pressure simulations there was a decrease in the w_s_ of the larger size classes (>237 and above for the present pCO_2_ conditions, >280 and above for the future pCO_2_ conditions). For both the present and simulated atmospheric conditions, the decrease in larger particle sizes was accompanied by an increase in the smaller size classes. Pressure had a significant effect on the w_s_ of the 8 smallest size classes: >63 µm (p = 0.002), >75 µm (p = 0.024), >88 µm (p = 0.046), >104 µm (p = 0.017), >122 µm (p = 0.004), >144 µm (p = 0.011), >170 µm (p = 0.002), >201 µm (p = 0.008), and of the largest size class (>460 µm, p = 0.034). The pCO_2_ had a significant effect on the ws of the aggregates >104 µm (p = 0.04), >122 µm (p = 0.031), >280 µm (p = 0.033), >331 µm (p = 0.007) and >390 µm. The interaction of pressure and pCO2 had a significant effect on the ws of aggregates >63 µm (p = 0.018), >201 µm (p = 0.047), >237 µm (p = 0.047) and >280 µm (p = 0.008). Settling velocity was normally distributed for all group combinations of pCO_2_ and pressure, as assessed by the Shapiro-Wilk test (p>0.05). There was homogeneity of variances, as assessed by the Levene Test of Homogeneity of Variance (p>0.05).

## Discussion

### PIC and POC

The influence of hydrostatic pressure on the dissolution of PIC in the ocean has been previously calculated [25], and some experimental data exist on the effect of hydrostatic pressure on the dissolution-precipitation of calcite [26] and aragonite [27]. Our experiments confirmed an enhanced dissolution of PIC in aggregates under increasing hydrostatic pressure. The increased PIC dissolution under acidified conditions means that, in future acidified oceans, there will be an increased release of alkalinity in an earlier stage of the aggregate flux, at shallower depths than at present, due to the dissolution of carbonates. This effect can slow down the vertical transfer of alkalinity, effectively reducing a net source of atmospheric CO_2_ [28].

In contrast, differences in POC degradation were larger under conditions of atmospheric pressure. These results suggest that the degradation of POC within the twilight zone was reduced under enhanced hydrostatic pressure. An inhibition of the bacterial community of surface aggregates under enhanced hydrostatic pressure has been previously described [29], [30], [31], and is potentially an important mechanism in the preservation of organic matter during fast vertical transport. Additionally, the degradation of POC was intensified under simulated future pCO_2_ conditions. This suggests that more acidified future oceans will increase the rate of dissolution of organic matter in the upper water column. This would increase the pCO_2_ of the upper layers, which would affect the equilibrium with the atmosphere, thus slowing the uptake of atmospheric CO_2_ [32].

### Particle Size

Approximately 75% of the particles were larger than 280 µm, both before and after the incubation. These largest size classes were more affected by the simulated environmental conditions of increased pCO_2_ and pressure, with the higher loss of particle numbers occurring in the 390 and 460 µm size classes. This suggests that a disaggregation process occurred with these larger aggregates. This disaggregation resulted in a relative increase of the 280 and 331 µm size classes. This disaggregation process can be an effect of the higher rates of inorganic carbon dissolution observed, which would alter the mineral matrix of the aggregate. Changes in mineral matrix have been observed to alter the size of aggregates [33]. Based on that study [33] the increase of mineral load would lead to a disaggregation, with a reduction in size of the aggregates. Thus we would not expect a disaggregation as consequence of the reduction of ballast. However, the overall alumino-silicate load of the system remained constant during the experiment, which might imply a greater role of carbonates in establishing the cohesion of larger aggregates. This was noted by Engel et al [34], who observed that the presence of CaCO_3_ stimulated aggregation processes in phytoplanktonic aggregates and resulted in larger and faster sinking aggregates. The loss of carbonates would then result in enhanced disaggregation.

### Settling Velocity (w_s_)

Under future pCO_2_ conditions, the size classes of 280 and 330 µm show a reduction in settling velocity. This result is also in accordance with the results of Engel et al [34] and can be related to the decrease in CaCO_3_ and disaggregation of larger aggregates. These size classes increased as a percentage of total particles due to the disaggregation of larger particles. As seen above, this disaggregation can be related to the loss of CaCO_3_ ballast, which would also explain the reduction in settling velocity. This decrease of the settling velocity would consequently slow the flux of matter to the deep-sea because the settling velocity of an aggregate is directly related to the amount and quality of organic matter that will resist degradation during the descent [12], [13]. Such a decrease in the settling speed would result in enhanced residence times during which aggregates could be colonized by barophilic bacteria and degraded [31]. The statistical analysis indicates that this effect is dependent both on the pCO_2_ conditions and the hydrostatic pressure. This suggests that in a future acidified ocean aggregates formed at the surface would settle slower, and consequently be more effectively degraded than in the present ocean.

### Implications

The CO_2_ exchanges between the atmosphere and the ocean are biologically mediated, and depend on the “rain ratio” [13], [35] which is the ratio between the production and vertical export of POC and the production and vertical export of PIC. This ratio will affect the pH of deeper waters, determining the long term capture of CO_2_ [35]. It is not clear from our data how the rain ratio will be affected in future oceans, since there are competing mechanisms at play. On the one hand, the vertical export of PIC will be reduced due to a shallower dissolution of carbonates. On the other hand, there will be an elevated degradation of POC, which the consequent reduction of settling velocity due to loss of ballast will further enhance. Nonetheless, and independently of the rain ratio, overall lower amounts of labile organic carbon would be exported to the deep-sea communities via benthic pelagic coupling. Our results also suggest that studying these phenomena solely under atmospheric pressure will underestimate the effects on the size and settling velocity of the aggregates, while overestimating the export flux of the organic matter.

### Conclusions

This experiment has shown that future pCO_2_ conditions can significantly change the ballast composition of sinking aggregates. The change in aggregate composition in turn shifts the size distribution of the aggregates, and the shift to smaller aggregates leads to a higher residence time of the aggregates in the water column, where it can be continuously degraded. In the environment, such an effect would result in a reduction of the carbon flux to the deep-sea. This reduction would impact those benthic communities, which rely on the vertical flow of carbon as primary source of energy. This effect will be felt in the beginning of the 22^nd^ century, or – if projections continue to prove conservative – even in the later part of the 21^st^ century. It will possibly pose a great threat to biodiversity in these communities, especially in areas at high latitudes, where the impact of temperature increase and drop of pH will already be felt in deeper waters.

This experiment also shows the need for the use of experimental setups that assure realistic conditions of hydrostatic pressure, when studying the effects of ocean acidification in deeper waters.
